# Ubiquitous Computing in Sports and Physical Activity—Recent Trends and Developments

**DOI:** 10.3390/s22218370

**Published:** 2022-11-01

**Authors:** Arnold Baca, Peter Dabnichki, Che-Wei Hu, Philipp Kornfeind, Juliana Exel

**Affiliations:** 1 Centre for Sport Science and University Sports, University of Vienna, 1150 Vienna, Austria; 2 STEM College, RMIT University, Melbourne, VIC 3000, Australia

**Keywords:** pervasive computing, cloud computing, wearable technology, sports performance, physical activity, performance analysis

## Abstract

The use of small, interconnected and intelligent tools within the broad framework of pervasive computing for analysis and assessments in sport and physical activity is not a trend in itself but defines a way for information to be handled, processed and utilised: everywhere, at any time. The demand for objective data to support decision making prompted the adoption of wearables that evolve to fulfil the aims of assessing athletes and practitioners as closely as possible with their performance environments. In the present paper, we mention and discuss the advancements in ubiquitous computing in sports and physical activity in the past 5 years. Thus, recent developments in wearable sensors, cloud computing and artificial intelligence tools have been the pillars for a major change in the ways sport-related analyses are performed. The focus of our analysis is wearable technology, computer vision solutions for markerless tracking and their major contribution to the process of acquiring more representative data from uninhibited actions in realistic ecological conditions. We selected relevant literature on the applications of such approaches in various areas of sports and physical activity while outlining some limitations of the present-day data acquisition and data processing practices and the resulting sensors’ functionalities, as well as the limitations to the data-driven informed decision making in the current technological and scientific framework. Finally, we hypothesise that a continuous merger of measurement, processing and analysis will lead to the development of more reliable models utilising the advantages of open computing and unrestricted data access and allow for the development of personalised-medicine-type approaches to sport training and performance.

## 1. Introduction

Pervasive or ubiquitous use of computing, i.e., embedding sensor information, processing and communication technology into everyday objects, has been integrated into scores of sports and exercise-related areas. Conspicuous examples include scientific experiments in motion studies conducted under valid ecological conditions; assistive technology helping recreational and health-conscious athletes in their physical activities; and performance and tactical analyses of association football games providing analysis immediately after the end of a match, just to name a few.

In an earlier review paper on ubiquitous computing technologies in sport from 2009 [1], the authors concluded that “… the emphasis in the future developments will shift to the development of intelligent systems that could not only analyse the data but suggests strategies and interventions”. As 13 years have passed, we scrutinise whether this prediction has become a reality by analysing current practices and future trends. 

We discuss the state of the art in micro-electro-mechanical system inertial measurement units (MEMS IMU) and current approaches for data acquisition on human activities and sports. The merging of these sensors and cloud computing technologies is explored later in the work, while we first discuss the advantages of the wearable devices that, in combination with mobile computing, have gained a strong foothold in sport performance analysis.

**Figure 1:**
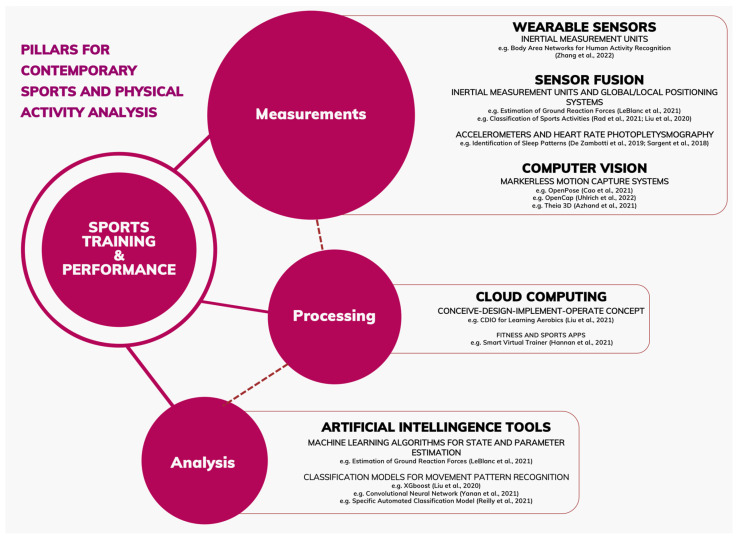
Current methods applied to the analysis of training and performance of sports and physical activity-related issues. Examples from the measurement methods described are in Zhang et al. [2], LeBlanc et al. [3], Rad et al. [4], Liu et al. [5], De Zambotti et al. [6], and Sargent et al. [7]. For methods of processing, current examples are described in Liu et al. [8] and Hannan et al. [9]. Finally, for analysis methods, examples are described in LeBlanc et al. [3], Yanan et al. [10], and Reilly et al. [11].

We consider a number of ubiquitous computing applications in sports performance and health. Among these applications are the developments in computer vision with deep learning algorithms used to assess sports skills, to promote injury prevention and to provide key performance indicators in several sports. The advancements in the integration of different sensors in wearable intelligent monitoring systems are broadly outlined. In particular, sensor fusion is utilised in sports and health monitoring to quantify exercise parameters and body response, to provide classification of activities and movement patterns, to estimate energy expenditure and to assess sleeping patterns. Using selected examples, we set out the current state of the art in the outlined areas, as depicted in Figure 1, and make conjectural predictions and recommendations for future development.

## 2. Sensors and Measurements in Human Activities and Sport

Sensor development has influenced all areas of life, from personal and business communications to home appliances and broadcasting. While sport and fitness have rarely been the driving force behind sensor development, sport performance analysis and sport science in general have benefited from the reduction of sensor size and the consequent increase in the ability to gather simultaneous measurements. This sea change was brought about by the adoption of technologies such as MEMS (micro-electrical-mechanical systems) that allow a multitude of measurements in a compact size unit of low weight. This technology has been underpinned by the immense progress in wireless transmission crucially complemented by a technological breakthrough in the development of long-lasting, compact, and low-weight power supply units. This development allowed for the pervasive propagation of this technology to all echelons of sport, from the fitness enthusiast up to top-level professionals. What seems to have been overlooked is that single-measurement sensors have been mass-produced since the last decade of the 20th century. It suffices to mention Polar heart rate monitors (Polar Electro Oy, Kempele, Finland), a must-have gadget for sport enthusiasts at the time. 

The most visible advance is the introduction of the inertial measurement units (IMU) in sport, a specific form of MEMS that allows data collection from multiple sources in relation to the dynamics of human motion (a standard unit comprises of accelerometer, gyroscope and magnetometer for the gravity direction). However, some of them target outdoor sports and also collect temperature and environmental data. The big difference in comparison to the wearable devices of the past is that this technology is not adapted from other medical applications but was developed for traditional engineering measurements, and its implementation in sport poses certain new challenges. While IMUs are accurately calibrated for engineering hardware, where different modal frequency responses could be identified either by direct measurement or using highly accurate predictive models, this issue is not so easy to address in sport/health/rehab devices. Wearable IMUs provide human motion data that are used as input to models, but human models do not possess the same degree of accuracy due to higher complexity and variability in the response of biological structures and general movement patterns. 

A recent work [2] employs miniature sensor devices within low-energy-consuming body area networks (BAN) for human activity recognition (HAR). This approach has been implemented in various applications such as daily life assistants, medical treatment, sports analysis. The HAR systems collect data predominantly from wearable sensors while data processing and analysis are performed on a host machine. The drawback of this approach is the necessity for real-time transmission and sensor data processing. This particular investigation focuses on the co-design of hardware to meet the software requirements and in turn provides optimal outcomes from a wearable HAR sensor network. The stated contributions include (1) design and use of integrated MEMS IMU for non-invasive direct sensing; (2) smart data processing utilising in situ computation; and (3) optimised classification algorithms using artificial intelligence techniques. While experimental studies were conducted with two sensor nodes worn on the wrist and elbow to verify the effectiveness of the recognition of 10 virtual handwriting activities, they really lack sufficient complexity to justify the stated achievements. Not surprisingly, it is claimed that the proposed system is reported to deliver an accuracy of 99%, which is statistical certainty and suggests overly simplified classification.

The discussed works clearly demonstrate the advantages of hardware–software fusion with the increased analytical power brought by artificial intelligence to provide broad pattern classification. The authors also avoided the pitfall of going into individual analysis of subjects’ performance patterns and variability. Their approach also underlines the limitations of the current tendency of group observations—the uniqueness of the individual performance is “smudged” in the broad classification. In terms of sport performance, it means that the approach is appropriate from basic to intermediate level and cannot be expanded. The more interesting question is: where is the source of these limitations? We outline the main ones below:

1. The data provided by the IMUs is already pre-processed—it means smoothed and altered, as significant deviations are “cleaned”. The approach is perfectly reasonable for population-type studies, but high-level athletes are stunted individuals as they are more than three standard deviations from the mean and our sensors remove vital information about their idiosyncratic features. This feature may in equal measure hold the secret for superior performance by a highly talented and unique individual or be an early sign of injury and/or wear. Consequently, the predictive ability of any intelligent system is curtailed.
2. The location of the sensors within the BAN is based on loose instructions and basic ergonomic assumptions. The data processing is built on assumptions for accurate positioning, which is in practice impossible to achieve, and hence the outputs are post-processed to only produce realistic motion patterns. Once again, an AI approach takes over the accuracy requirements and uses statistical corrections that necessarily eradicate unusual individual characteristics.
3. Analysis of variability requires highly personalised models that allow to follow individual deviations which necessitates longitudinal continuous studies that are only possible with high-level dedicated funding. However, the hidden danger in this approach is that data are unlikely to be made publicly available due to privacy issues. As a result, data analysis and subsequent decision making is unlikely to be subjected to peer review and more importantly is not supported by an appropriate level of statistical power. Still, the development of personalised models requires both investment and open sharing to speed up their development.

The presented discussion points out certain limitations of the current approach, but its validity is not questioned and by no means undermined. Simply, statistical models are highly effective in population studies but not appropriate for small-scale individual studies. Still, the personalised medicine approach, which is a different type of statistical approach that employs multi-parametric analysis, could be a highly effective tool and currently forms the backbone of the artificial intelligence applications in sport and health.

## 3. Cloud Computing

Cloud computing defines an expanded use and delivery model of Internet-based services. The method comprises frequent transactions with remote resources, fast exchange and secure storage in sometimes virtual servers. Cloud computing offers economical scalability, while it breaks barriers to knowledge transfer and exchange. The CDIO concept (conceive—design—implement—operate) is a concept ideally suited to flourish within cloud computing technology [8] as the CDIO teaching model combines theory and practice based on an open system. The concept has spread into all fields of education. The CDIO teaching model has been utilised for teaching aerobics in colleges. CDIO is a key concept that needs to be fine-tuned to deliver effective talent training. In its current form, it represents a conceptual platform/repository containing different educational modules. The quoted paper researches and analyses the application of the CDIO teaching mode in college sports aerobics under the background of cloud computing.

Fitness and sport apps and devices have drawn considerable attention to wearable and pervasive computing. As a result of the breakthrough in wireless technology, standard mobile phone service facilitates the connection between the wearable devices and cloud computing not only by large companies but also by smaller commercial entities [12]. Physical activities are without a doubt essential for improved health and general well-being, but frequently the desire for over-achievement and/or lack of knowledge on safe performance may cause temporary or even permanent disability. As a result, injury prevention is a major area of research and development of smart monitoring systems. In the new reality of COVID, where fitness is widely performed at home rather than in shared spaces such as traditional gyms, smart portable fitness sites are highly desirable [9]. The particular system alerts via Android app when inappropriate posture is assumed and actively encourages posture correction. A k-nearest neighbours (KNN) classification model guides the user when performing a particular exercise. Overall, the system seems a good step towards smart virtual trainers but the tests produced are narrow in scope. 

The next work addresses another conundrum—can clients use the technology to self-direct and optimise their training program? Jang and Lv [13] aimed to design a sports training system by using data from a wireless sensor network. The implemented arrangement of sensor nodes and motion database software collects, stores and analyses the user’s motion parameters. Finally, the practical tests of the wireless sensor network-based sports training suggest that the system is appropriate for sports training use, but the optimisation of the training routines is still not definitive.

The last two works effectively illustrate the capabilities of the new technology and the advancements of sport science and medicine. However, a word of caution is needed, i.e., all these systems are usually tested on healthy young enthusiasts over a relatively short period. The observation is not related to these works but is a general practice in fitness research and even more so in consumer product development, where longitudinal population representative studies are seldom conducted. In practice, while early gains are relatively easy to achieve, their effect is usually short-lived for numerous reasons, not least that the data are usually collected in isolation, and there are unobserved factors affecting the individual’s health and fitness level. Furthermore, trends are notoriously unreliable, especially at the individual level and users’ adherence to best practices is rarely guaranteed. As a result, the feel-good factor wears down and users tend to drop the systems and replace them with the next novelty [14]. 

Following this brief overview, we would like to summarise that modern MEMS, cloud computing and AI tools have made tremendous progress in the last 10–15 years and reached wider audiences. Most technology issues have been addressed and education systems have become highly effective and reliable. However, tailoring such systems to individual needs requires a much higher degree of customisation based on highly reliable predictive models, which are not available yet. The focus of the future work needs to be on longitudinal studies where not only group performance is considered but individual response as well. The computational power allows researchers to assess multiple factors, such as pre-existing conditions, past incidents, and other factors, to allow effective intelligence building for more reliable trend prediction. The adaptation patterns also need to be better understood and further investigated.

## 4. Applications in Performance and Health in Sports

Athlete development and performance preparation are currently driven by the interaction with the environment as a performance regulator [15]. Thus, the design and application of tools enabling the learning and training processes should foster a more ecological approach and ought to be user-centred. In the late 2000s, ubiquitous computing relied heavily on the combination of computers with sensors and transmitting devices to track players in sports games [1], while most decision-making systems were purely experimental. Current technological and scientific advances are sufficiently mature to materialise automated decision-making systems in both training and competition environments.

### 4.1. Computer Vision: Markerless Motion Capture Technology

Markerless motion capture systems and algorithms have been constantly improved in the past 5 years. Modern computer vision algorithms utilising neural networks have been adapted for assessing movement patterns in sports performance and physical activity providing researchers practical means for faster data analysis with ecological validity, i.e., outside the restrictive environment of labs. An example of a freely accessible software is OpenPose, which is the most widely used tool for pose estimation. OpenPose is open-source, allows for tracking of human body landmarks and has also been used as part of algorithms estimating joint centres for kinematic analysis by markerless motion capture systems [16]. 

Gait analysis is the most explored application, and the development of markerless technology has provided sophisticated means of running technique performance and injury prevention/rehabilitation analyses. Different OpenPose-based algorithms have been proposed to estimate segment and joint kinematics from either standard digital cameras [17] or smartphone images [18], and its performance was compared against 3D marker-based motion capture data (MoCap). Results have shown differences of less than 1° in joint angle estimation for hip and knee [13]. Gait parameters such as speed, cadence, step length and step time showed mean absolute differences lower than 2% [18]. Ota et al. [19] applied OpenPose to obtain segment and joint angles during walking and running, also comparing data obtained through a MoCap, and although the measurements in the sagittal plane corresponded, the movement in the frontal and transverse planes rotation for the pelvis and hip showed substantive disagreement. Theia3D (Theia Markerless Inc., Kingston, ON, Canada) is another deep learning-based algorithm for markerless 3D pose estimation. This system delivered spatiotemporal parameters across multiple sessions in gait that were reliable and comparable to MoCap. Kanko et al. [20,21] found in running analysis that the average root mean square (RMS) distance between corresponding joint centres was less than 3 cm, except for the hip (<4 cm), as lower limbs appeared more sensitive to gait cycle compared to the upper limbs. 

Sports injury is another fundamental topic that has been under the radar of markerless system development. The ability to objectively and accurately predict risk factors and assess the readiness of an athlete after surgery or any other intervention is critical. Mauntel [22] used a dedicated software to process the depth data via proprietary kinematic machine learning algorithms. Better agreement was found in the kinematics of the trunk, hip and knee in the sagittal plane in comparison to frontal plane peak angle values during the landing phase but not for peak values at initial ground contact of the jump landing and joint displacement outcomes.

In general, markerless kinematics enhances researchers’ capabilities for ecological validity of sport performance analysis and allows for a broader field of application. In association football, for instance, lower limb kinematics is the determinant for kicking performance and seems to have found support from markerless systems. Automatic estimation of hip, knee, ankle and foot kinematics was obtained through OpenPose and compared to manual image digitisation during kicking tasks performed outdoors, showing to be a reliable approach to movement in sagittal and frontal planes [23]. However, movement velocity and foot–ball contact might increase the errors associated with the estimated kinematics. Needham et al. [24] proposed and assessed a computer vision method based on pose estimation to provide accurate estimates of body centre of mass (CoM) kinematics of athletes and the sled during skeleton bob races. MoCap and OpenPose data capture were synchronised for assessment and comparison of sprint start and skeleton push start stride characteristics and mass centre velocities for both athlete and sled. Alpine skiing is another sport that could and should benefit from markerless technology. Ostrek et al. [25] proposed a monocular computer video-based approach to estimate 3D pose and ski orientation from 2D images, also comparing the data to a MoCap system, on a typical giant-slalom course, with high-definition single-view cameras. In both studies, the number of cameras and their specific setup on the volume of interest were crucial to the results obtained; thus, reproducing the methods might be challenging. Additionally, in the case of the computer vision applications, the amount of available training data used to validate the methods should still be improved to background better accuracy and precision of the estimates. 

In addition to kinematic analysis, OpenCap was proposed to evaluate 3D kinematics and kinetics of human movement [26]. It is an open-source, web-based simple-to-use software application that guides users through a fast setup for data collections but requires a minimum of two iOS devices (iPhone^®^, iPad^®^ or iPod^®^) placed on tripods, a calibration checkerboard and a third device to run OpenCap’s web application. Kinematic characteristics are derived using OpenPose and HRNet algorithms as well as inverse kinematics in OpenSim, while kinetics is obtained via a physics-based musculoskeletal simulation. The software was validated against MoCap and force plate data in healthy individuals during squatting and jumping. Mean absolute errors (MAE) of averaged joint rotations were 4.1° and 5.1°, respectively, and of joint centre translations, they were 12.3 mm and 11.15 mm, respectively. The MAE for the vertical ground reaction force and joint moments for both movements were between 6.4% and 25.2% of the body weight. The authors also assessed the accuracy of estimating joint loads with a view for injury and disease prevention, as knee moments, and predicted the early-stance peak knee adduction moment with a correlation coefficient of 80% to MoCap and force plates. It is promising, but OpenCap still needs further development in the deep learning model and optimisation formulation of the simulations to be generalisable to other tasks and activities.

Markerless systems have demonstrated some potential to assess sports performance related kinematics, as could be seen in the works related in the previous paragraphs. Inevitably, there are challenges to better utilise markerless systems in sports or physical activity. The main limitation lies in the accurate reconstruction of movements in the transverse plane, as internal and external rotations consistently show the lowest agreement values when compared to the gold standard MoCap. Difficulties in reconstructing joint centre positions are mostly associated with how well the proposed algorithms detect body landmarks, which in turn depends on the number and positioning of cameras and dataset size used for training the machine learning algorithms in each specific scenario/sport/condition. Most approaches target not only the automation of movement reconstruction in space but also aim to reduce the necessary equipment. This approach ultimately results in the omission of some important performance parameters; thus, one might question the trade-off limit between data reliability and equipment reduction. Solutions involving multi-body optimisation methods such as OpenSim and OpenPose have been proposed and shown to increase the robustness of joint angle calculations in gait modes such as walking, cycling and running [27,28], so that might be a path to overcome image reconstruction constraints. It should be pointed out that the fact that most solutions are free and open source enables knowledge sharing and data exchange, and quicker product development while offering open, uninhibited usage with lower equipment investment thresholds.

### 4.2. Sensor Fusion: Wearable Intelligent Monitoring Systems

Wearable sensor fusion has been a steady trend in the development of intelligent monitoring systems. Particularly, the combination of accelerometer, gyroscope, magnetometer—as an inertial measurement unit (IMU)—and global/local positioning (GPS/LPM) have been the most common type of sensor fusion with applications in sports performance and health. 

Quantifying and monitoring training and performance loads are crucial for the delivery of evidence-based decision making in athlete management [29]. Recent developments in IMU’s applications enable modelling and evaluation of vertical ground reaction forces (vGRF), tissue loading, and impacts as non-invasive measures for the encountered internal loads. Le Blanc et al. [3] used an IMU in the sacrum to estimate the body CoM motion and continuously estimate and track vGRF during running from a mass-spring-damper model, in which parameters such as leg stiffness are estimated to provide performance and injury-related information. Because vGRF estimation during running is non-linear and depends on biomechanical parameters that vary with fatigue, structural adaptation, running surface conditions and shoe, the method applies a combined dual-Kalman filter for state and parameter estimation, because of its online adjustment capabilities. This framework was compared against data acquired from an instrumented treadmill. The method performed well in estimating peak vGRF and cadence, in contrast to vertical impulse and the loading rate of the vGRF obtained until 40% of stance phase. 

An approach using multiple commercial sensors to estimate musculoskeletal stress in lower limbs during running, through the peak tibial compression force, is presented in Matijevich et al. [30]. IMU units were attached to the shank and foot and, combined with pressure-sensing insoles, yielded input data to a model based on the classic inverse dynamics approach in biomechanical modelling. A model based on machine learning algorithms using over 10 discrete features based on preliminary data exploration was developed. The results were normalised by participants’ body weight (BW) and compared against data acquired using synchronised MoCap and instrumentalised treadmill. The classic approach presented an RMS error of 0.5 BW, while data yielded from the machine learning algorithm presented a 0.25 BW RMS error in peak tibial force estimates. The authors concluded also that machine learning algorithms utilising combined pressure insole and foot IMU data improves peak tibial force estimates. However, adding shank IMU had a minimal effect on the RMS magnitude. It seems that an important step would be to understand to what extent the number of sensors significantly changes the accuracy of measurements related to sports performance. Two-IMU and one-IMU configurations were tested against MoCap system data in wheelchair sport-specific sprinting and ability tests [31]. The MAE for both approaches was 0.1 ms^−1^ for the linear velocity but higher than 8°·s^−1^ for the angular velocity with the single-device approach. There is a trade-off between the number of sensors and accuracy, and decisions should be made according to the conditions one might have to conduct data collection and determine which variables would answer one’s question.

The works mentioned above are interesting examples of recent IMU usage in sports and health applications but do not take advantage of its main feature, which is the fusion of various sensors into a unit device. In reality, only acceleration data were used, when sensor fusion has greater potential to assist the classification of human activities. One example is in the detection of performance-related swimming kinematics using the information provided by the sensors of a single IMU [4]. All signals were used to detect the different swimming phases in the four swimming styles and to derive performance kinematic metrics related to velocity and time and then compared to the same metrics extracted from the video images and speedometer. Still, if there is an interest in more detailed information on technical aspects of swimming, i.e., joint kinematics during the execution of the styles, this method is too simplistic and would not fulfil the desired outcomes. Further development in the classification of limb techniques in swimming would be necessary.

Finally, the potential of IMUs to assess body orientation in sports movements was also evaluated in comparison to MoCap data [32]. Researchers applied an algorithm to compute an optimised fusion of trunk and pelvis orientation estimates by minimising the effect of these errors on the accuracy of IMU orientation. Sports movements, such as golf swing, one-handed ball throw, tennis serve and baseball swing, as well as isolated trunk motions, were recorded. It was found that up to 95% of the variance found in the data collected by the MoCap system could be explained by trunk angles from the IMUs during the dynamic sports movements, with an RMS error of 5° between systems in all planes of motion.

The classification of human activities in sports performance can be improved when the sensors’ signals are used as input for machine learning algorithms. Recently, the ability of algorithms in distinguishing such datasets based on different conditions have been tested on canoeist’s level of expertise [5], different actions in table tennis [10], types of kick in Australian football [33] and change of directions in association football [11]. Together with the use of multiple fused sensors, a recent work has also tested different machine learning algorithms to recognise movement patterns. In Liu et al. [5], the XGBoost algorithm accurately recognised 98.5% of the different stroke phases when using the selected features in canoeing. Yanan et al. [10] used a convolutional neural network and fine-grained evaluation with a refined segmentation of the hitting attitude data of table tennis movements. Algorithms performed very differently in the identification, characterisation, and keyframe extraction representative of all stroke phases. Cust et al. [33] applied different classification models to detect Australian football 2-Kick and 4-Kick at different distances (10, 20, 30 m). The results of overall accuracy for 2-Kick and 4-Kick classification were higher (over 80%) when the random forest model was applied, while kick distances were more difficult to estimate with the classification models used (overall accuracy of 63%). Reilly and colleagues [11] developed an automated classification model to assess movements with changes of direction during competitive matches in association football. Players wore a multi-sensor device containing an augmented navigation satellite system and IMUs to provide position and attitude, and the random forest classification model was applied. The classifier showed a high ability to identify change of direction movements, with an area under the curve of 0.96; however, data showed a dependence on the threshold for the classification used on the model sensitivity and specificity for correct/incorrect classification rates in changes of direction. Interestingly, the variables related to accelerometry had the same predictive power to identify changes of direction in association football as those yielded by the gyroscope. The improvements of classification models in sport are still being challenged by the variability of movement patterns, which impacts directly the quality and quantity of the available training dataset.

Sensor fusion has been established as an alternative for obtaining kinematic parameters with reasonable accuracy in field environments. IMUs are a popular example and show some promise to substitute MoCap in some specific situations. Most studies in sports performance, though, still make use of only acceleration (resultant acceleration, in most cases) to calculate variables related to performance, so the full capacity of this type of sensor is not explored as a tool for movement analysis. The fact is that it is still not possible to appraise the applications of sensor fusion in sports in a broader overview. The use of combined signals to assess different movement aspects is a work in progress. As already mentioned, the development of algorithms for signal processing and data analysis requires further development to become a feasible technology for sport performance assessment. The identification of daily life activities demonstrates that merging multiple sensors, big data processing techniques and the Internet of Things into an automated recognition system delivers some interesting results. Wu et al. [34] present the ADL Recorder App, a client application for smartphones, which collects data from the embedded microphone, Wi-Fi module, IMU, light proximity, step detector, etc. to measure daily living ability through activities such as eating, bathing, getting dressed, toileting, studying and shopping. With the help of datasets and application programming interfaces available for researchers, future developments of sports-related recognition systems could evolve further.

Monitoring and assessing sleep patterns and their relationship with sport performance are debated topics in training and sports sciences. Sleep is a crucial ingredient in athletes’ recovery strategies and is associated with overall health, cognitive functioning and academic and sport performance [35]. Wearables have also been used as sleep trackers, and the embedded algorithms are based either on accelerometer data to estimate sleep intervals via the frequency and intensity of activity, or on accelerometers and heart rate photoplethysmography [6]. Such monitors demonstrated capabilities to accurately estimate sleep time but require considerable development to automatically detect sleep/awake periods. Accelerometer-only-based wearables such as FitBit (HR Charge; Fitbit Inc., San Francisco, CA, USA) purport 80% detection success for night-time sleep periods of 7–9 h and only 30% for daytime naps of 1–2 h. Total sleep time can be overestimated for 4 to 5 h sleep [7] when compared to polysomnography (PSG), which is the gold standard measure for the structure and depth of sleep. Another device, the WHOOP strap (Generation 2.0, CB Rank, Greater Boston, MA, USA), when tested against PSG, overestimated sleep and wake periods by 8 min, with a confidence interval around sleep bias of ±64 min and an overall agreement of 89%. Other commercially available trackers have also been tested. ACTICAL Z series (ACTICAL; Mini-Mitter Philips Respironics, Inc., Bend, OR, USA) and WHOOP strap were tested against PSG. ACTICAL and WHOOP presented over 90% capacity for identifying sleep correctly but 60% for wake. ACTICAL overestimated total sleep time by 38 min and underestimated wake time by −38 min, and no differences between WHOOP and PSG were found [36]. Most studies use participants’ reports as a control for sleep/awake periods to assess athletes and healthy people. Nonetheless, sleep disorders, such as obstructive sleep apnoea syndrome, have also been a target of wearable intelligent monitoring systems. Hernandez and Cretu [37] proposed an embedded system solution to monitor the respiratory effort and body position in real time during sleep, using a single IMU with an extended Kalman filter for data fusion. Although applied to a limited sample, the method showed a high correlation (96%) to a piezoelectric belt used as a reference (the variations in length or tension on the belt reflect the activity of the respiratory muscles during the breathing cycle).

The important limitation of current sensors and embedded algorithms is their ability to automatically detect the landmark time points of sleep (i.e., REM), which should be addressed to achieve objective detection independent from user-entry information. Furthermore, access to raw data is restricted and should be made available to accelerate the development of automated systems. The commercially available wearables do now allow users to export data in epochs, or even the raw data. This limits the depth of analysis and control of the measurements.

## 5. Conclusions and Future Directions

The work analyses recent advancements in hardware and the application of ubiquitous computing, predominantly in sports and physical activity. Selected examples are used to illustrate identified trends as a comprehensive review is not conducted. In summary, novel approaches that have been proposed, tested and made available in the past 5 years show advancements in the type of biological signals able to be collected in field/ecological settings. Kinematic-related (position, acceleration, angular velocity, attitude) and cardiovascular responses to exercise and sport (heart rate) are the main examples of current data obtained by wearables, that are used in a multitude of models to assess overall health and fitness status with varying degrees of reliability. There is a substantive step forward in the development and implementation of cloud computing solutions to acquire, store, process and share data and provide assessments. IMUs are also very popular among sensor fusion approaches to improve the classification of human activities and external mechanical load measurements. While most studies are successful in presenting neat data for population-type studies, high-level athletes’ samples still find some difficulties in terms of signal processing that can deal with the details that are away from centrality descriptors and vital information as individual responses are still removed as part of data smoothing routines. Thus, reliable predictive models are needed to address those needs and are expected to be available in the near future. In the universe of new applications for movement analysis, for example, markerless systems have been efficiently worked towards automatic detection and calculation of kinetics and kinematics. The detection of the CoM of the body or sports implements represent great advancements in computer vision. Still, there is a general difficulty in enhancing the detection of positions in certain planes, such as the transversal plane.

We would like to summarise that sensor technology maturity is now sufficient to address the analysis needs at all sport levels from casual participant to professional athlete. However, for the higher performance level cohorts, more sophisticated models are needed that are based on substantive cohort sizes. This task cannot be performed using the standard approach of recruiting participants for a short-term study as, for example, top-level athletes do not usually reside at the same place and their performance is dependent on the time point in their training cycle. The advent of cloud computing and open-source software allow for the accumulation of data and knowledge and allow for the formation of virtual cohorts that do not perform concurrently, have protected identity and benefit from the enhanced understanding of their abilities and temporal limitations that lead to performance improvement, extended longevity and fewer injuries. We foresee the first stage to be the development of reliable decision support systems that benefit both athletes and their support personnel.
